# Sindbis Virus–Host Interactions in Human Neuroblastoma Cells: Implications for Viral Pathogenesis and Replication

**DOI:** 10.3390/v17101346

**Published:** 2025-10-07

**Authors:** Kornélia Bodó, Zoltán Kopasz, Viktória Nyári, Krisztina Leiner, Péter Engelmann, Brigitta Zana, Roland Hetényi, Dániel Hanna, Krisztián Bányai, Mónika Madai, Gréta Varga, Anett Kuczmog

**Affiliations:** 1 National Laboratory of Virology, Szentágothai Research Center, University of Pécs, H-7624 Pécs, Hungary; bodo.kornelia@pte.hu (K.B.); kopasz.zoltan@pte.hu (Z.K.); nyari.viktoria@pte.hu (V.N.); leiner.krisztina@pte.hu (K.L.); zana.brigitta@pte.hu (B.Z.); roland.hetenyi@aok.pte.hu (R.H.); daniel.hanna@aok.pte.hu (D.H.); madai.monika@pte.hu (M.M.); varga.greta@pte.hu (G.V.); 2 Department of Immunology and Biotechnology, Medical School, Clinical Center, University of Pécs, H-7624 Pécs, Hungary; engelmann.peter@pte.hu; 3 RoLink Biotechnology Kft., Szentágothai Research Center, University of Pécs, H-7624 Pécs, Hungary; 4 Department of Medical Biology, Medical School, University of Pécs, H-7624 Pécs, Hungary; bkrota@hotmail.com; 5 Department of Pharmacology and Toxicology, University of Veterinary Medicine, H-1078 Budapest, Hungary; 6 Institute of Biology, Faculty of Sciences, Department of Molecular Biology and Microbiology, University of Pécs, H-7624 Pécs, Hungary

**Keywords:** SINV, viral replication, UV-C inactivation, apoptosis, immune modulation

## Abstract

Sindbis virus (SINV) is a mosquito-borne alphavirus capable of causing neurological and immunological symptoms in humans, yet its effects on neural/immune systems remain insufficiently characterized. This study aimed to examine SINV replication, UV-C light inactivation, apoptosis induction, and immune gene modulation in human SH-SY5Y neuroblastoma cells. Following viral adaptation and infectious dose determination, SINV replication and inactivation were assessed using RT-qPCR and dsRNA immunofluorescence. Apoptotic markers (caspase-3, Bax, Bcl-2) were analyzed by immunofluorescence and immune genes expression kinetics (*TLR3/7*, *RIGI*, *MDA5*, *IL-1β*, *IL-6*, *TNFα*, *IL-10*, *IFNβ* and *β-catenin*) were measured at defined time points post-infection by RT-qPCR. SH-SY5Y cells supported productive SINV infection, with viral RNA detectable as early as 3 hpi and marked cytopathic effects by 24 hpi. A custom-built UV-C chamber achieved complete viral inactivation following 3 × 30 s exposures. We observed SINV time-course replication and UV-C inactivation with conspicuous morphological alterations in SH-SY5Y cells. Furthermore, SINV triggered caspase-dependent apoptosis and robust transcriptional upregulation of innate immune genes, peaking between 12–16 hpi and declining by 30 hpi. These findings elucidate the temporal dynamics of SINV replication, cell death mechanisms, and immune activation in a neuronal context, contributing to a better understanding of SINV neuropathogenesis.

## 1. Introduction

The Sindbis virus (*Alphavirus sindbis*, SINV) (*Togaviridae*) is an emerging pathogen with significant epidemic potential across Eurasia, Africa, and Oceania. Maintained in an enzootic cycle involving avian hosts and mosquito vectors (*Aedes*/*Culex species*), its geographic range is expanding due to factors like climate change and globalization. Although human infections are typically associated with fever, rash, and polyarthritis, SINV is also neurotropic and capable of invading the central nervous system (CNS). As a consequence, SINV infection can lead to a range of neurological manifestations, including encephalomyelitis (inflammation of the brain and spinal cord), paralysis, and behavioral changes [1,2]. The absence of approved vaccines or specific antiviral therapies underscores the urgent need to understand its pathogenesis, particularly the molecular and cellular interactions within neural tissues.

A critical aspect of SINV neuropathogenesis is the host-specific innate immune response within the CNS. Neuronal cells are not passive targets; they possess intrinsic defense mechanisms to detect and combat viral threats. This response is initiated by pattern recognition receptors (PRRs), such as Toll-like receptors (TLRs) and RIG-I-like receptors (RLRs), which recognize viral components. Activation of these PRRs triggers signaling cascades that lead to the production of antiviral molecules (e.g., type-I interferons), pro-inflammatory cytokines (CKs) (e.g., interleukins (ILs)) and tumor necrosis factor-alpha (TNF-α) [1,3,4]. However, an excessive or dysregulated immune response can contribute to neuroinflammation and tissue damage, highlighting the need for a balanced regulatory network involving anti-inflammatory mediators, such as IL-10 and signaling pathways such as Wnt/β-catenin [5,6,7,8].

Beyond the immune response, a key outcome of alphavirus infection in the CNS is the induction of programmed cell death, or apoptosis. This process is tightly controlled by a balance between pro-apoptotic proteins (e.g., Bax, caspase-3) and anti-apoptotic proteins (e.g., Bcl-2). Virus-induced apoptosis can serve as a host defense mechanism to limit viral spread, but it can also lead to significant neuronal loss and contribute to the neurological symptoms of the disease [9,10]. The interplay between these antiviral, inflammatory, and apoptotic pathways ultimately determine the fate of the infected neuron and the pathological outcome in the host. Understanding these dynamics is crucial for developing targeted neuroprotective and antiviral interventions [11]. One possible solution could be the usage of ultraviolet (UV) light for viral inactivation, which has emerged as a promising and cost-effective strategy for vaccine development. UV-inactivated viruses retain their structural integrity and immunogenicity while losing infectivity, enabling the induction of robust immune responses without the risks associated with live-attenuated vaccines. Such approaches offer potential not only for broad immunization efforts but also for protecting vulnerable tissues such as the CNS from virulent viral strains [12].

Compared to *Chikungunya virus* (CHIKV), the molecular and immunological mechanisms of SINV infection remains less well characterized. While SINV-induced neuronal dysfunction and cell death are strongly suspected—mirroring findings from related alphaviruses—comprehensive studies are still lacking and necessary to explain the underlying pathogenesis. To dissect these complex interactions, this study utilized the human SH-SY5Y neuroblastoma cell line as an in vitro model for neuronal infection. Our primary objective was to provide a comprehensive, time-resolved analysis of the cellular and molecular events following SINV infection. Specifically, we aimed to (i) characterize the kinetics of SINV replication and its associated cytopathic effects, (ii) establish an effective UV-C light-based protocol for viral inactivation, providing a safe tool for future immunological studies, (iii) investigate the activation of the intrinsic apoptotic pathway by analyzing the expression of key cellular markers, and (iv) quantify the transcriptional dynamics of crucial immune-related genes, including *PRRs*, *CKs*, *antiviral effectors*, *and immunoregulatory genes*. Together, these findings can help us to better understand the neuropathogenesis of SINV-associated CNS infection in humans.

## 2. Materials and Methods

### 2.1. Virus and Cell Line

A human neuroblastoma cell line, SH-SY5Y (ATCC^®^ CRL-2266, Manassas, VA, USA), cloned originally from a metastatic bone tumor, was used for all in vitro experiments. Cells were cultured at 37 °C in a humidified atmosphere containing 5% CO_2_ in Dulbecco’s Modified Eagle Medium (DMEM; Sigma-Aldrich, St. Louise, MO, USA), supplemented with 10% fetal bovine serum (FBS; Gibco, Waltham, MA, USA), 1% MEM Non-Essential Amino Acids (NEAA; Sigma-Aldrich, St. Louise, MO, USA), and 1% penicillin-streptomycin (PS; Lonza, Basel, Switzerland).

The viral strain used in this study was SINV (UVE/SINV/2017/DZ/P29). Viral stocks were propagated in Vero E6 cells, a kidney epithelial cell line derived from the African green monkey (*Cercopithecus aethiops*) (ATCC^®^ CRL-1586™, ATCC, Manassas, VA, USA), and stored at −80 °C until use. For infection assays, culture medium was replaced with infection medium containing 2% FBS, 1% NEAA, and 1% PS.

### 2.2. TCID_50_ Assay for Virus Titration

The tissue culture infectious dose 50 (TCID_50_) assay was performed to determine the infectious titer of SINV strain based on cytopathic effect (CPE) in SH-SY5Y cells. SH-SY5Y cells were seeded at a density of 5 × 10^4^ cells per well in 96-well tissue culture plates (TPP, Sigma-Aldrich, St. Louise, USA) and allowed to adhere overnight at 37 °C in a humidified incubator with 5% CO_2_. Serial 10-fold dilutions of the virus were prepared in infection medium (DMEM supplemented with 2% FBS, 1% NEAA, and 1% PS), and 100 µL of each dilution was added to eight consecutive wells in a column. After 24 h of incubation at 37 °C, wells were examined for CPE using an inverted Primovert HD Cam microscope (Carl Zeiss Inc., Jena, Germany), with particular attention to subtle morphological changes indicative of infection. The TCID_50_ endpoint was determined, and focus forming units per milliliter (FFU/mL) were estimated using the formula: FFU/mL = (number of foci or infected cell clusters)/(dilution factor × volume of diluted virus). Based on the calculated FFU/mL, the multiplicity of infection (MOI) was determined. MOIs of 1 (used as a positive control in antibody-based assays), 0.005, and 0.001 (applied in gene expression assays) were selected for downstream experiments. All procedures were conducted under appropriate biosafety conditions in accordance with institutional and national guidelines.

### 2.3. Viral RNA Isolation, Quantitative Reverse Transcription (RT-qPCR) and Copy Number Determination

SH-SY5Y cells were seeded into 24-well plates (Sarstedt, Nümbrecht, Germany) at a density of 2.5 × 10^5^ cells/well and incubated overnight at 37 °C in a humidified atmosphere with 5% CO_2_. On the following day, cells were infected with SINV at MOI of 0.005 and 0.001 for various time points (3, 6, 12, 16, 24, and 30 h), using infection medium. Appropriate virus dilutions were made and SH-SY5Y cells were exposed for 1 h. Uninfected cells served as negative control. After 1 h, the virus was removed and replaced with fresh supplemented culture medium. Following the designated incubation periods, cells were harvested by centrifugation (1500 rpm, 5 min), and the majority of the supernatant was removed. Viral RNA was subsequently extracted from both the residual supernatant and the cell pellet using the Quick-RNA Viral Kit (Zymo Research, Cambridge, UK), according to the manufacturer’s protocol. Eluted RNA samples were stored at −80 °C until further processing. Viral RNA quantification was performed using the Luna^®^ Universal Probe One-Step RT-qPCR Kit (New England Biolabs, Ipswich, MA, USA). The primer sequences were as follows: SINV_NSP1_F: 5′-TGA TAC TGG TGC GAA AAC A-3′; NSP1_R: 5′-GGT TCC TAC CAC AGC GAC GAT-3′; and the probe: FAM-5′-TTG GAC ATA GGC AGC GCA-3′ [13]. Thermal cycling was conducted on a CFX Opus Dx Real-Time PCR System (Bio-Rad, Hercules, CA, USA) with the following program: 1 cycle at 55 °C for 10 min (reverse transcription), 1 cycle at 95 °C for 1 min (initial denaturation), followed by 40 cycles of 95 °C for 10 s and 60 °C for 30 s. To generate a standard curve for absolute quantification, viral cDNA was first purified by gel electrophoresis and extracted using the DNA Gel Extraction Kit (New England Biolabs, Ipswich, MA, USA). DNA concentration was then measured with a Qubit dsDNA BR Assay Kit (Thermo Fisher Scientific, Invitrogen, Waltham, MA, USA) according to the manufacturer’s instructions. Serial ten-fold dilutions of the purified cDNA were prepared and included in each RT-qPCR run to determine viral copy number, similarly than we performed earlier [14].

### 2.4. UV-C Inactivation of SINV and Testing Its Infectivity

A custom-built ultraviolet (UV) irradiation chamber (RoLink Biotechnology, Pécs, Hungary) was used to inactivate the virus stock. The inactivation process was carried out in transparent, non-autoclaved, 1.5 mL centrifuge tubes, each containing 1 mL of virus suspension (4.6 × 10^5^ FFU/mL). The tubes were placed individually into the UV chamber, constructed of stainless steel and equipped with a 11 W mercury UV lamp emitting UV at a wavelength of 253.7 nm. During a 3 × 5 s, 3 × 15 s and 3 × 30 s irradiation period, the virus suspension received a direct UV-dose of approximately 100 mJ/cm^2^ from the lamp, supplemented by an additional 20 mJ/cm^2^ reflected from the chamber’s walls. Hereafter, UV-dosage is indicated as irradiation time in seconds, with 30 s corresponding to an approximate total UV dosage of 120 mJ/cm^2^. Complete inactivation was confirmed using TCID_50_, passaging and PCR assays. For infectivity testing, 200 µL of UV-inactivated SINV was transferred to columns of a 96-well tissue culture plate (TPP, Sigma-Aldrich, St. Louise, MO, USA) containing SH-SY5Y cells (5 × 10^4^). The cells were then incubated for 3 days at 37 °C in a humidified CO_2_ incubator. After the incubation period, 100 µL of the supernatant was transferred to a new plate that had been seeded with fresh SH-SY5Y cells (5 × 10^4^). Then, 100 µL of fresh culture medium was added. This passaging process was repeated twice, resulting in 3 infection rounds total. Viral-RNA was isolated at each passage (including the initial time point, referred to as passage 0) using a standard RNA extraction protocol and analyzed via RT-qPCR to assess residual viral RNA (as we described above, M&M 2.3.). The dilutions were correctly adjusted for RNA analysis. Remaining aliquots of UV-inactivated SINV were stored at −80 °C for future immunofluorescence staining experiment (M&M 2.5.).

### 2.5. Identification of Viral Replication and Apoptotic Markers by Immunofluorescence

SH-SY5Y cells were seeded at a density of 2 × 10^5^ cells/well into 8-well glass chamber slides (Biologix, St. Louise, MO, USA, Cat# 07-2108) and incubated overnight at 37 °C in a humidified atmosphere containing 5% CO_2_ to allow cell adherence. On the following day, prior to infection, the supernatant was gently replaced to remove non-adherent (floating) cells. Virus dilutions were prepared from the SINV stock, and cells were infected for 1 h. Uninfected cells served as negative controls. Following the infection period, the virus was removed and replaced with fresh, supplemented medium. Cells were then incubated for an additional 24 h. The next day, cells were fixed with ice-cold methanol for 30 min. For blocking, a 1% bovine serum albumin (BSA; Thermo Fisher, Waltham, MA, USA) solution in phosphate-buffered saline (PBS) was applied for 30 min, as described previously [14]. For immunofluorescent staining, various primary antibodies (Abs) were applied to detect viral and apoptotic markers. The following Abs were used: mouse anti-double-stranded (ds)RNA monoclonal antibody J2 (mAb; Nordic Mubio, Susteren, The Netherlands, Cat# RNT-SCI-10010200, 1:1000), rabbit anti-Caspase-3 polyclonal antibody pAb (Thermo Fisher, Waltham, MA, USA, Cat# PA1-26426, 1:200), rabbit anti-Bax pAb (Thermo Fisher, Waltham, MA, USA, Cat# PA5-11378, 1:100), and rabbit anti-Bcl-2 pAb (Thermo Fisher, Waltham, MA, USA, Cat# PA5-27094, 1:200). Cells were incubated with primary Abs for 1 h at room temperature (RT) in the dark. After washing, secondary Abs were applied: Goat Anti-Mouse IgG H&L Alexa Fluor^®^ 488 (Abcam, Cambridge, UK, Cat# ab150113, 1:1000) or Goat Anti-Rabbit IgG H&L Alexa Fluor^®^ 488 (Abcam, Cambridge, UK, Cat# ab150077, 1:1000), depending on the host species of the primary antibody. Secondary antibody incubation was carried out for 1 h at RT in the dark. Nuclear staining was performed using DAPI (4′,6-diamidino-2-phenylindole) solution (Invitrogen, Waltham, MA, USA, Cat# D1306) for 10 min at RT. Between each staining step, cells were washed five times with PBS as previously described. Initial fluorescence images were acquired using a Nikon ECLIPSE Ti-U Series inverted fluorescence microscope (Nikon, Tokyo, Japan). For high-resolution imaging and detailed subcellular analysis, samples were further examined with the same settings as before using a Zeiss LSM 710 confocal laser scanning microscope (Carl Zeiss Inc., Jena, Germany) equipped with Plan-Apochromat objectives (10×/0.45 NA, 20×/0.8 NA, and 63×/1.4 NA). Imaging parameters—including laser intensity, filter settings, and pinhole size—were standardized across all samples. Low-magnification images were obtained using an open pinhole, while optical sectioning was performed at 0.5 μm intervals for high-magnification z-stacks [14].

### 2.6. Total RNA Isolation, cDNA Synthesis and qPCR-Based Quantification of Immunological Markers Expression

SH-SY5Y cells were seeded and infected for the same time points as described above (M&M 2.3.). The only modification was that, following centrifugation, the entire supernatant was removed prior to RNA isolation. Each infection condition was performed in biological triplicates. Then, total RNA was extracted from SH-SY5Y cells using Direct-zol RNA MiniPrep RNA isolation kit (Zymo Research, Cambridge, UK) according to the manufacturer’s protocol. The total RNA was measured using Qubit RNA BR Assay kit (Thermo Fisher, Invitrogen, Waltham, MA, USA) according to the provided protocol. The reverse transcription (25 °C for 2 min, 55 °C for 10 min, 95 °C for 1 min) was performed by LunaScript RT Super Mix Reverse Transcription Kit (New England Biolabs, Ipswich, MA, USA) according to the standard protocol. For each reaction, 500 ng of total RNA was reverse transcribed in a final volume of 20 μL. The resulting cDNA was stored at −20 °C until further use. For SYBR Green-based quantitative PCR (qPCR), cDNA templates were mixed with gene-specific primer pairs designed using Primer Express software 3.0 (Thermo Scientific, Waltham, MA, USA). Primer sequences (PRRs (*RIG-I*, *MDA-5*, *TLR-3*, *TLR-7*), inflammatory (*IL-6*, *IL-1β*, *TNFα*), -anti-inflammatory (*IFN-β*, *IL-10*)—and regulator (*β-catenin*) genes) are listed in Supplementary Table S1. Gene expression levels were quantified using the CFX Opus Dx Real-Time PCR System (Bio-Rad, USA) in combination with Brilliant III Fast SYBR qPCR Master Mix (Agilent Technologies, Santa Clara, CA, USA). The amplification profile started at 95 °C and lasted for 3 min, followed by steps throughout 40 cycles at 95 °C for 10 s and 60 °C for 30 s. Dissociation curve analysis of amplified products was performed at the end of each reaction to confirm the generation of a single PCR product. *RPLP0* mRNA level was implemented for normalization. Gene expression level analysis was performed as we described earlier [15,16]. All reactions were performed in technical duplicates, and data were independently evaluated for consistency.

### 2.7. Statistical Analyses

Statistical analyses were carried out by GraphPad Prism 5.0 (GraphPad Software, Boston, MA, USA). qRT-PCR data were evaluated using two-way ANOVA followed by Šídák’s multiple comparisons test and Tukey’s multiple comparison post hoc test to determine the significance of the data. All results represent the mean and standard error of the mean (±SEM). Differences were considered statistically significant at *p* < 0.05.

## 3. Results

### 3.1. SINV Infection Results in Marked Morphological Alteration in SH-SY5Y Cells

**Figure 1:**
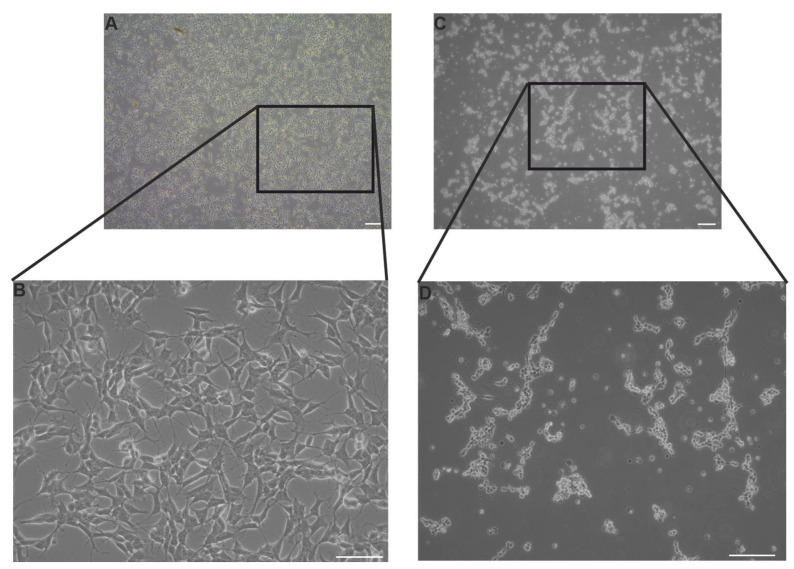
Representative light microscopy images of SH-SY5Y cells under control conditions and following 24 h of SINV infection at MOI 1. (**A**,**B**) Uninfected (CTRL) SH-SY5Y cells; scale bars: 100 μm (**A**) and 50 μm (**B**). (**C**,**D**) SH-SY5Y cells infected with SINV; scale bars: 100 μm (**C**) and 50 μm (**D**).

Morphological examination of control and SINV-infected SH-SY5Y cells revealed clear differences within 24 hpi (Figure 1). Representative phase-contrast images of control SH-SY5Y neuroblastoma cells demonstrated their characteristic neuroblast-like morphology, which is marked by compact cell bodies and extended neurite-like projections. These cells exhibited strong adherence, uniform spatial distribution across the culture surface, and intact cellular membranes, without signs of blebbing or detachment (Figure 1A,B). Contrastingly, SINV-infected SH-SY5Y cells exhibited pronounced cytopathic effects, including cell shrinkage, membrane blebbing, and detachment from the culture substrate (Figure 1C,D), clearly differing from the healthy morphology of control cells. The TCID_50_ assay revealed the extent to which dilutions should be prepared for gene expression studies. The choice of MOI 0.005 and 0.001 (calculation explained above in M&M, 2.3.) was based on these values because our goal was to observe how the virus induces changes in cells despite its small amounts. In the long term this would result in significant morphological alterations and extensive cell death (Figure S1).

### 3.2. Sindbis Virus Replication Dynamics Are Strongly Influenced by Initial MOI

**Figure 2:**
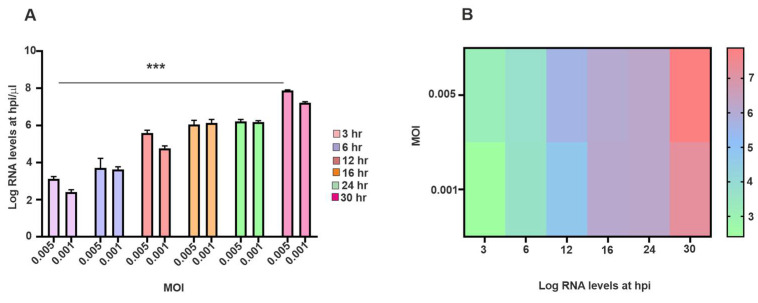
Detection and kinetics of SINV nucleic acid in SH-SY5Y cells. (**A**) Viral copy numbers were quantified using a TaqMan-based RT-qPCR assay following infection with SINV at MOI of 0.005 or 0.001. Cells were harvested at multiple time points between 3- and 30-hpi. Viral RNA quantities are expressed in log_10_ values. Data represent mean ± SEM from three independent experiments (*n* = 3). Statistical significance: *** *p* < 0.001. (**B**) The temporal distribution of viral copy numbers is illustrated by a heat map.

To evaluate the effect of the initial input of virus on SINV replication dynamics, a time-course TaqMan-based RT-qPCR analysis was conducted on SH-SY5Y cells, which were infected at two different MOIs (0.005 and 0.001) (see Figure 2A). Viral RNA levels were quantified at multiple time points post-infection to monitor the dynamics of replication. As shown in Figure 2, viral RNA accumulation displayed a clear MOI-dependent pattern. At MOI 0.005, an early and exponential increase in RNA levels was detected starting at 6 hpi, peaking by 24 h. In contrast, the MOI 0.001 condition demonstrated a delayed onset of viral RNA detection and consistently lower RNA levels during early and intermediate time points. Nevertheless, it is undeniable that both viral inputs could establish infection, as viral RNA was detectable as early as 3 hpi, with levels increasing significantly over time (Figure 2A). The accompanying heat map illustrates the temporal distribution of viral RNA, where the relative signal intensity corresponds closely with the qPCR quantification (Figure 2B). These results underscore that a higher MOI accelerates and amplifies SINV replication dynamics. Statistical analysis confirmed a significant effect of both MOI (*p* < 0.01) and time (*p* < 0.001) on viral RNA accumulation. Importantly, significant differences between MOI 0.005 and 0.001 emerged from 12 hpi onward (*p* < 0.05), highlighting a cumulative impact of the initial viral dose on replication efficiency over time.

### 3.3. Detection of dsRNA in SH-SY5Y Cells as an Indicator of Viral Replication

**Figure 3:**
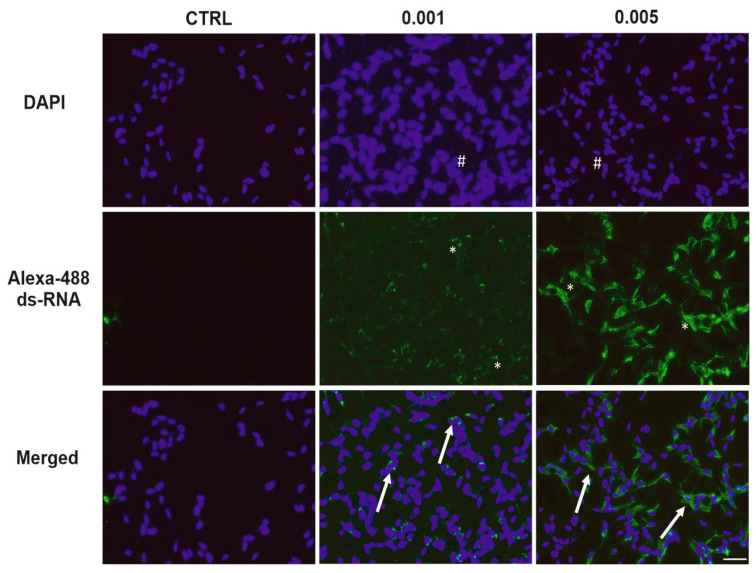
Detection of dsRNA in SH-SY5Y cells to determine viral replication. Fluorescence immunocytochemistry was used to visualize dsRNA-positive cells 24 hpi of SINV at MOI 0.005 and 0.001. Uninfected cells served as controls (CTRLs). Cell nuclei were stained with DAPI (blue), while dsRNA-positive cells appear green. Merged signals (arrows) indicate colocalization of nuclear and dsRNA signals. Asterisks mark dsRNA-positive cells. Number signs: nuclei (blue). Scale bar: 50 μm.

To determine the presence of viral replication intermediates, SH-SY5Y cells were immunolabeled with an anti-dsRNA Ab following infection with SINV at two different MOI values (0.005 and 0.001) (Figure 3). No dsRNA signal was detected in the control (uninfected) samples, indicating the specificity of the Ab-based staining. In contrast, SINV-infected cells exhibited a clear, distinct dsRNA signal localized to the cytoplasm consistent with active viral replication (Figure S2). The intensity and amount of prevalence of dsRNA-positive cells were notably higher at an MOI of 0.005 compared to 0.001, in line with the increased viral load expected at higher infection doses. Furthermore, nuclear staining with DAPI revealed similar cell densities under all conditions, indicating that variations in the dsRNA signal were not attributable to variations in cell number (Figure 3).

### 3.4. SINV Inactivation Was Successful upon 3 × 15 and 3 × 30 s UV-C Exposure

**Figure 4:**
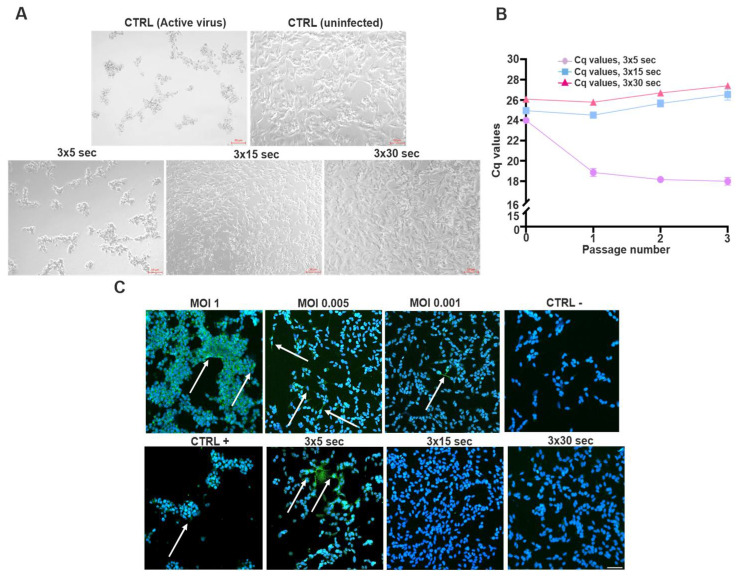
UV-C inactivation of SINV and detection of viral nucleic acids using RT-qPCR and immunofluorescence across serial passages. (**A**) SINV was subjected to UV-C inactivation using three sequential exposure durations: 3 × 5 s, 3 × 15 s, and 3 × 30 s. Following each treatment, the virus was used to infect SH-SY5Y cells. After a 3-day incubation period, supernatants were collected and used to infect fresh SH-SY5Y cultures for a total of 3 serial passages. Prior to each passage, representative light microscopy images of the cells were captured (scale bar: 50 μm). (**B**) To assess the presence of residual or replicating viral RNA, a TaqMan-based RT-qPCR assay was performed on samples from each condition (*n* = 3 ± SEM). Visualization of Cq values accurately indicates the presence of only degraded viral RNA or its replication despite UV exposure. (**C**) (arrows) To further confirm the effectiveness of UV inactivation, immunofluorescence staining was employed to detect dsRNA-positive cells—a hallmark of active viral replication. The same UV-C exposure durations (3 × 5 s, 3 × 15 s, and 3 × 30 s) were applied, as previously described. For comparison, different MOI values were also included. After the third passage, immunostaining was also performed to detect the virus’s RNA. Scale bar: 50 μm.

To evaluate the effectiveness of UV-C inactivation of SINV, the virus was exposed to UV-C irradiation for 3 × 5, 3 × 15, and 3 × 30 s durations, respectively. The treated viral preparations were subsequently employed to infect SH-SY5Y cells, followed by 3 consecutive passages (Figure 4). Microscopic examination was conducted prior to each passage, and representative images were captured to document potential CPE. The results revealed that a 3 × 5 s exposure was insufficient for complete viral inactivation, as the infected cells displayed pronounced cytopathic changes like those observed with active virus. In comparison, UV-C exposure for 3 × 15 and 3 × 30 s did not induce any observable cytopathic changes, and the morphology of the treated cells was indistinguishable from uninfected controls (Figure 4A). To further assess the presence of viral replication, TaqMan-based RT-qPCR was performed to quantify viral RNA levels. The obtained Cq values reflected either residual, non-infectious viral RNA or ongoing replication following UV treatment. Notably, lower Cq values showed that a 3 × 5 s UV-C exposure was insufficient to prevent viral replication. In contrast, prolonged exposures (3 × 15 and 3 × 30 s) resulted in higher Cq values, consistent with effective inactivation and lack of productive infection. These findings suggest that complete inactivation, preventing subsequent viral replication, is achieved at exposure durations of 3 × 15 s or longer (Figure 4B). dsRNA-positive cells were predominantly observed under conditions involving shorter UV-C exposure durations, supporting the RT-qPCR results and indicating ongoing viral replication. In contrast, extended UV-C treatments (3 × 15 or 3 × 30 s) markedly reduced or eliminated dsRNA immunoreactivity, confirming effective viral inactivation (Figure 4B,C).

### 3.5. Apoptotic Pathway Is Induced to SINV Infection

**Figure 5:**
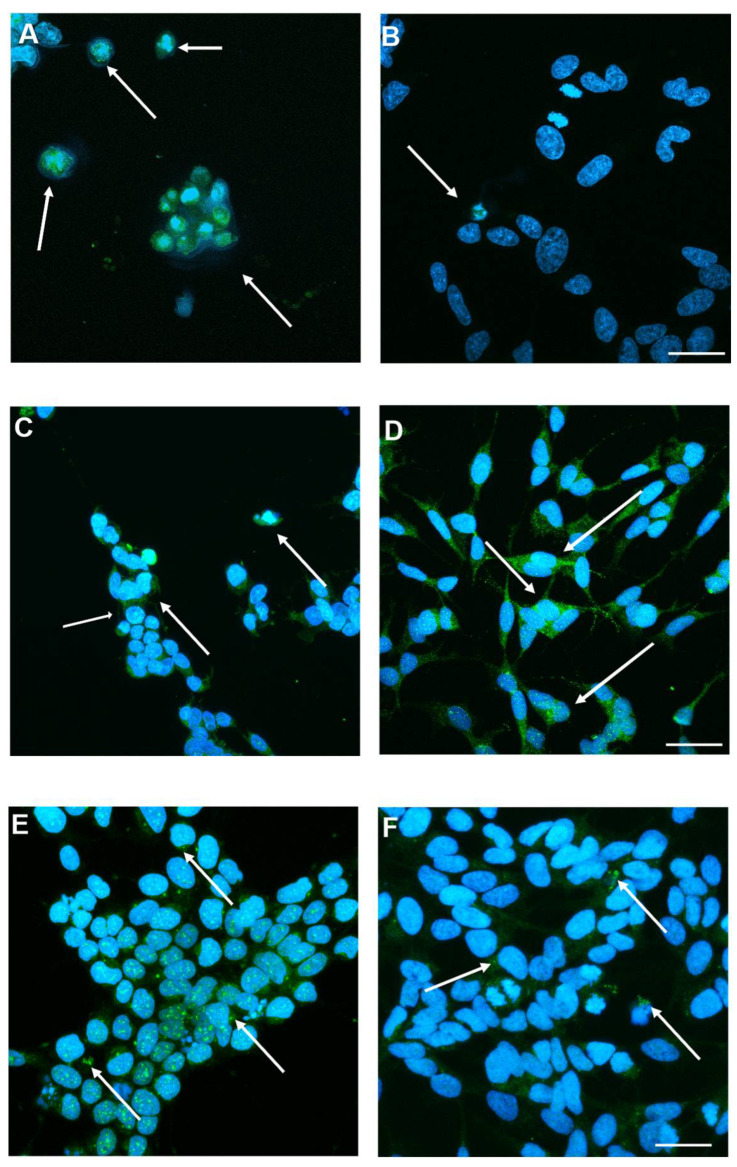
Detection of apoptotic or anti-apoptotic protein markers following SINV infection. (**A**) Representative images show the presence of activated caspase-3 in cells infected with MOI 1 and (**B**) MOI 0.005, and (**E**,**F**) expression under similar conditions at higher magnification. (**C**) Anti-apoptotic Bcl-2 was detected in both SINV-infected cells (MOI 1) and (**D**) uninfected CTRL cells. Arrows indicate (**A**,**B**) caspase-3 positive cells, (**C**,**D**) Bcl-2 positive cells or (**E**,**F**) Bax positive cells, highlighting differential protein expression in response to SINV infection. Scale bar: 20 μm.

We examined the expression and subcellular localization of apoptotic and anti-apoptotic proteins in SH-SY5Y cells in response to SINV infection at varying MOIs. Figure 5A and Figure 5B demonstrate the immunodetection of the pro-apoptotic effector caspase-3 following infection with MOIs of 1 and 0.005, respectively. Consistent with Figure S3, caspase-3 immunoreactivity was also observed in cells infected with MOI 0.001 and in non-infected control samples, although at markedly reduced levels. The presence of caspase-3 positive cells is suggesting that apoptosis is induced following SINV infection. Increased caspase-3 expression is more prominent at the higher MOI, indicating a dose-dependent apoptotic response (Figure 5A,B), suggesting that SINV infection triggers caspase-mediated neurodegeneration in the CNS. Figure 5C,D show the immunolocalization of the anti-apoptotic protein Bcl-2 in SH-SY5Y cells following SINV infection at MOI 1 and in uninfected control cells, respectively. The detection of Bcl-2 in both conditions indicates its constitutive expression; however, potential differences in intensity or localization may suggest a response to viral infection. Figure S4 demonstrates Bcl-2 labeling across all tested MOIs (0.001, 0.005, and 1), with notably higher signal intensity observed at lower MOIs compared to MOI 1. However, their labeling intensity was significantly enhanced in comparison with MOI 1. Infected cells exhibited a marked reduction in Bcl-2 signal intensity at MOI 1, relative to control cells, suggesting that SINV infection suppresses anti-apoptotic signaling pathways (Figure 5C,D). This downregulation may contribute to a shift in the intracellular apoptotic threshold, favoring cell death over survival. Figure 5E,F present the immunodetection of Bax, another pro-apoptotic marker, in SH-SY5Y cells following SINV infection at MOIs of 1 (Figure 5E) and 0.005 (Figure 5F), respectively, under high-magnification imaging. Bax-positive cells, indicated by arrows, are more abundant under the MOI 1 condition. This reflects enhanced activation of apoptotic signaling pathways in response to an increased viral load. In addition to its cytoplasmic localization, Bax was also observed to accumulate in the nucleus, suggesting a potential role in nuclear apoptotic processes following SINV infection. This dose-dependent increase in Bax expression mirrors the pattern observed with Caspase-3 labeling, reinforcing the notion of a coordinated apoptotic response elicited by SINV infection (Figure 5E,F). Consistent with this, Figure S5 illustrates Bax immunoreactivity in also MOI 0.001 and control cells, where the signal is markedly reduced, further supporting the correlation between infection intensity and apoptotic activation. Given the neuronal lineage of SH-SY5Y cells, the upregulation of Bax suggests mitochondrial involvement in virus-induced neuronal apoptosis (Figure 5E,F and Figure S5).

### 3.6. Early Induction of PRRs Genes mRNA upon SINV Infection

**Figure 6:**
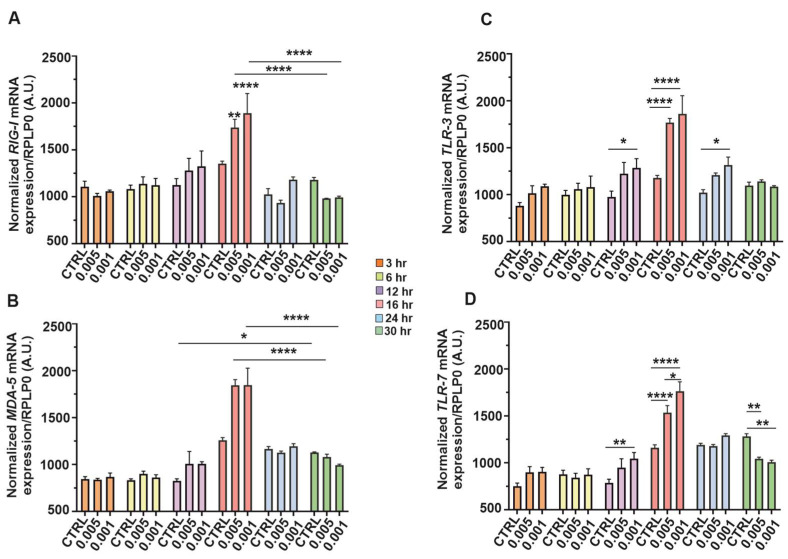
Temporal expression of *PRR* genes in SH-SY5Y cells in response to SINV challenge. The cells were infected with SINV at MOI of 0.005 and 0.001 in a timely manner. The expression patterns of the target genes were monitored over time (3, 6, 12, 16, 24, and 30 hpi) in SH-SY5Y cells during SINV infection. The temporal transcriptional profiles of (**A**) *RIG-I*, (**B**) *MDA5*, (**C**) *TLR3*, and (**D**) *TLR7* are depicted. The bar graphs represent the means of three independent biological replicates, each performed in technical duplicates. Error bars indicate the standard error of the mean (±SEM). Statistical significance was assessed using two-way ANOVA followed by Tukey’s multiple comparison test, performed with GraphPad Prism software (*n* = 3 ± SEM; * *p* < 0.05, ** *p* < 0.01, **** *p* < 0.0001). Gene expression levels were normalized to *RPLP0* mRNA levels.

Briefly, Figure 6 illustrates the temporal expression dynamics of key *PRR* genes in SH-SY5Y neuroblastoma cells following infection with low MOIs of SINV, specifically 0.005 and 0.001. Gene expression was monitored at multiple time post-infection (3, 6, 12, 16, 24, and 30 h), normalized to *RPLP0* mRNA, and compared to both control (uninfected) samples and between MOI conditions. Overall, an early induction of *PRR* expression was observed in response to SINV challenge, indicative of an innate antiviral response (Figure 6). In particular, *RIGI* (Figure 6A)*,* a cytoplasmic RNA sensor that plays a critical role in recognizing viral dsRNA showed a mild increase at 6 hpi, which became significantly elevated compared to the control by 16 h. However, a marked downregulation in *RIG-I* transcript levels was observed thereafter in both MOI conditions, ultimately falling below those of uninfected cells at later time points, potentially reflecting feedback inhibition or viral-mediated immune evasion (Figure 6A). Figure 6B illustrates the expression kinetics of *MDA5*, another cytoplasmic viral RNA sensor involved in antiviral responses. Similar to *RIGI*, *MDA5* follows a similar time-dependent upregulation, further supporting the functional engagement of cytosolic RNA sensing pathways in SH-SY5Y neuroblastoma cells upon SINV infection. Figure 6C focuses on *TLR3*, a PRR located in endosomal compartments that detects dsRNA. Compared to control cells, *TLR3* expression increased as early as 3 hpi and was significantly elevated at 12 and 16 h, followed by a decline at later time points. This suggests a transient but robust contribution of endosomal RNA sensing to the early antiviral response against SINV (Figure 6C). Figure 6D shows the expression profile of *TLR7*, a receptor specialized in recognizing single-stranded (ss)RNA within endosomes. *TLR7* expression progressively increased over time, following a similar trajectory to *TLR3* (Figure 6C), indicating its involvement in the recognition of SINV-derived RNA species (Figure 6D). Collectively, these data highlight a temporally coordinated activation of both cytosolic and endosomal RNA sensing pathways, emphasizing their critical role in mounting innate immune responses in human neuronal cells during alphavirus infection (Figure 6).

### 3.7. Induction of Inflammatory, -Antiviral, and -Regulator Genes mRNA upon SINV Infection

**Figure 7:**
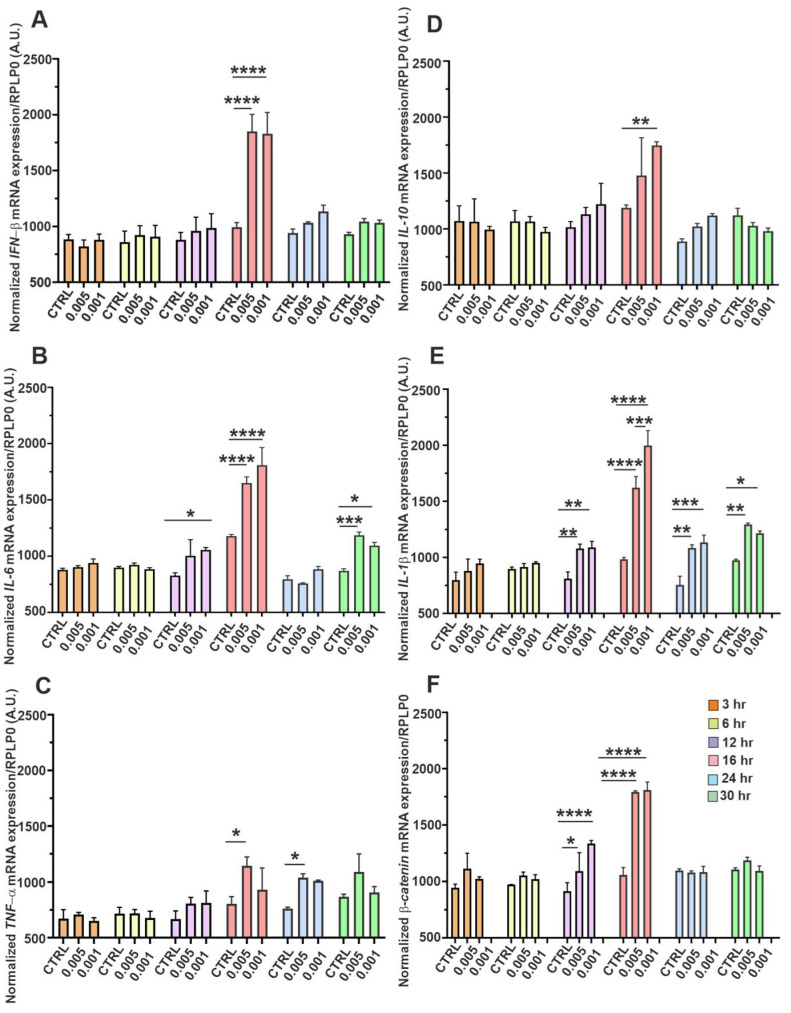
Temporal expression dynamics of antiviral, inflammatory, and regulatory genes in SH-SY5Y cells following SINV infection. SH-SY5Y cells were infected with SINV at MOI of 0.005 and 0.001. Gene expression was then assessed at 3, 6, 12-, 16-, 24-, and 30 hpi in a time-dependent manner. The panels display time-resolved mRNA expression patterns of key CK-s and regulatory molecules involved in the neuronal innate immune response: (**A**) *IFN-β*, (**B**) *IL-6*, (**C**) *TNF-α*, (**D**) IL-10, and (**E**) *IL-1β*, as well as (**F**) *β-catenin*, a gene implicated in immunomodulation and cell signaling. Bars represent the mean of three biologically independent experiments performed in duplicate, with error bars indicating the standard error of the mean (SEM). Statistical significance was determined using two-way ANOVA followed by Tukey’s multiple comparison test (GraphPad Prism; *n* = 3 ± SEM; * *p* < 0.05, ** *p* < 0.01, *** *p* < 0.001, **** *p* < 0.0001). Gene expression levels were normalized to *RPLP0* mRNA as a housekeeping reference.

We also measured the time-dependent expression profiles of key inflammatory CKs, antiviral markers, and a regulatory gene in SH-SY5Y neuroblastoma cells infected with SINV at low MOIs of 0.005 and 0.001 (Figure 7). Transcript levels were quantified at 3, 6, 12, 16, 24, and 30 hpi and normalized to *RPLP0* mRNA. Figure 7A displays the expression kinetics of *IFN-β*, a pivotal type-I IFN involved in antiviral defense. A progressive upregulation was observed over time, with a marked increase at later time points (notably from 16 hpi), suggesting a delayed but sustained activation of *type-I IFN* signaling pathways in response to SINV in neuronal cells. Figure 7B illustrates the temporal expression of *IL-6*, a pro-inflammatory CK implicated in neuroinflammation. Its expression followed an ascending trend, indicative of escalating inflammatory signaling as the infection progressed. Figure 7C shows the expression profile of *TNFα*, another central mediator of inflammation and apoptosis. Both *IL-6* and *TNFα* mRNA levels were significantly elevated from 12 hpi, with a pronounced peak at 16 h, highlighting the activation of pro-inflammatory pathways in response to SINV. Although their expression declined thereafter, levels remained elevated compared to uninfected controls even at 30 hpi, suggesting a sustained inflammatory environment in SINV-infected neuroblastoma cells (Figure 7B,C). Figure 7D presents the expression kinetics of *IL-10*, an anti-inflammatory CK that exhibits more moderate and potentially delayed induction. A noticeable elevation was observed by 12 hpi, reaching statistical significance at 16 h. However, by 30 h, expression levels returned to baseline, suggesting a transient compensatory response aimed at dampening excessive inflammation. Figure 7E shows the expression pattern of *IL-1β*, a potent pro-inflammatory CK known to contribute to neuroinflammatory processes. Like *IL-6* and *TNFα* (Figure 7B,C), *IL-1β* expression increased significantly after 12 h, peaking at mid-to-late stages of infection. While mRNA levels declined toward 30 hpi, they remained substantially elevated relative to control conditions, indicating persistent activation of inflammatory pathways in SINV-infected SH-SY5Y cells (Figure 7E). Finally, Figure 7F highlights the temporal expression of *β-catenin*, a multifunctional protein involved in Wnt signaling. Changes in *β-catenin* expression during infection suggest that SINV may disturb signaling pathways beyond classical antiviral and inflammatory responses, potentially, affecting broader regulatory mechanisms in neuronal-like cells (Figure 7F). Notably, differences in expression profiles between the two viral inputs (MOI 0.005 vs. 0.001) were consistently observed across multiple genes, indicating a dose-dependent modulation of host cellular responses. Collectively, this time-resolved expression analysis reveals the coordinated, yet complex, interplay among antiviral, pro-inflammatory, anti-inflammatory as well as regulatory gene networks in SINV-infected SH-SY5Y cells. These findings underscore the multifaceted nature of host-pathogen interactions in neuronal contexts and highlight the dynamic transcriptional landscape elicited by alphavirus challenge (Figure 7).

## 4. Discussion

Arboviruses are capable of causing a broad spectrum of human diseases. The geographic distribution of SINV is dynamic and reflects ecological and climatic drivers. Historically, outbreaks were endemic to northern Europe, Africa and parts of Asia. However, they have increasingly been reported in new regions, with seroprevalence data suggesting silent circulation in Oceania and the Middle East. More specifically, outbreaks have been reported in northern Europe, particularly in Finland, where recent epidemics suggest ongoing geographic expansion. In southern Europe, SINV has recently been isolated from mosquitoes in Spain, suggesting a recent introduction from Africa. The findings illustrate the role of migratory birds and mosquitoes in the long-distance dispersal of the virus. Furthermore, ecological and epidemiological studies indicate that changes in climate and the spread of vectors driven by globalization are expected to facilitate the further expansion of SINV transmission zones. These shifts highlight the epidemiological significance of SINV as an emerging pathogen with an increasing geographic and clinical impact. Additionally, in recent years, SINV has been associated with outbreaks marked by musculoskeletal symptoms, occasionally accompanied by neurological manifestations [3,4,17,18,19,20,21,22,23,24,25].

In the present study, we explored the effects of SINV infection on human SH-SY5Y neuroblastoma cells—a widely used in vitro neuronal model. We specifically investigated morphological alterations, viral replication dynamics, the expression of apoptotic and anti-apoptotic proteins, as well as the regulation of immune-related genes. Our findings offer new insights into SINV–neural cell interactions and the host’s cellular response, contributing to a deeper understanding of the virus’s neuropathogenic potential and immunological impact. The progression of SINV infection, along with the associated morphological alterations, demonstrated clear distinctions between infected SH-SY5Y cells and uninfected CTRLs, as illustrated by light microscopy and TCID_50_ assays (Figure 1 and Figure S1). These phenotypic changes were consistent with the observed dynamics of viral replication. Quantitative and qualitative assessments confirmed that SINV is capable of efficient replication in SH-SY5Y cells, as evidenced by increasing viral titers and mRNA levels over time (Figure 1 and Figure 2). The correlation between viral RNA accumulation and morphological disruption supports the notion that SH-SY5Y cells are permissive to SINV infection and can sustain productive viral replication (Figure 2 and Figure 3).

Previous studies have investigated the efficacy of UV-C inactivation in the context of CHIKV, a BSL-3 pathogen that requires strict biosafety measures. However, for many experimental approaches—especially those involving molecular or immunological analyses—handling under BSL-2 conditions is preferable. UV-C treatment, specifically 3 × 30 s exposures, was found to effectively inactivate CHIKV while preserving its antigenic properties, enabling its use in downstream applications under lower biosafety requirements [26]. In the current study, we applied a similar approach to SINV, a BSL-2 alphavirus, to determine optimal inactivation parameters. Our findings indicate that UV-C exposure durations of 3 × 15 s and 3 × 30 s were sufficient to halt SINV replication in SH-SY5Y cells, as confirmed by both molecular and imaging-based assays (Figure 4). These results not only support the feasibility of controlled SINV inactivation, but also lay the groundwork for future applications, including serological assay development and vaccine research, particularly in systems modeling neuronal infection [27].

It is well established that alphavirus infection can activate genetically encoded cell death pathways in host cells, most notably apoptosis. The induction of apoptosis is closely associated with characteristic morphological and biochemical hallmarks. Furthermore, it is influenced by both viral virulence factors and the intrinsic susceptibility of the host cell. More virulent alphavirus strains have been shown to trigger apoptotic responses more efficiently in both animal models and specific cell types. Importantly, the expression and activity of cellular genes involved in apoptotic regulation play a pivotal role in shaping the outcome of alphavirus infection, both in vitro and in vivo [4,28,29]. In the context of neuronal cells, such as SH-SY5Y neuroblastoma cells, the balance between pro-apoptotic and anti-apoptotic signaling is particularly critical, as dysregulation may contribute to virus-associated neuropathogenesis. Pro-apoptotic genes such as *caspase-3*, *Bax*, and *Bak* orchestrate and accelerate virus-induced cell death, whereas anti-apoptotic genes such as *Bcl-2* counteract this process, promoting cellular survival. While anti-apoptotic signaling may help preserve cell viability, it can also inadvertently support persistent viral infection. Thus, the cellular balance between pro, -and anti-apoptotic regulators plays a pivotal role in determining the outcome of viral infection, including that of SINV [5,28]. Previous studies have demonstrated that SINV exposure induces classical apoptotic features in neurons both in vitro and in the murine CNS [29]. Our findings align with these observations, indicating the activation of a caspase-dependent apoptotic cascade in SINV-infected neuronal-like cells. Specifically, we observed increased immunolabeling of caspase-3 (Figure 5A and Figure S3) and Bax (Figure 5C and Figure S5) after infection. This suggests the presence of mitochondrial-mediated apoptotic signaling. Notably, Bax immunoreactivity was not restricted to the cytoplasm; nuclear localization was also evident in several infected cells. This finding is consistent with previous reports indicating that Bax proteins are not confined to the cytoplasm or the endoplasmic reticulum but can also translocate to interphase nuclei and nuclear compartments, thereby expanding their apoptotic activity [30,31]. In contrast, higher levels of Bcl-2 staining were detected in control (uninfected) cells (Figure 5B and Figure S4), further supporting that SINV infection tilts the apoptotic balance toward cell death. Therefore, downregulation of Bcl-2 in infected cells may contribute to a shift in the intracellular apoptotic threshold, favoring cell death over survival. Considering the neuronal identity of SH-SY5Y cells, these findings underline the potential for SINV to modulate neuroprotective mechanisms, thereby enhancing neuronal susceptibility to apoptosis. The decreased Bcl-2 expression may thus represent a key molecular event in virus-induced neurodegeneration (Figure 5B and Figure S4). These results underscore the vulnerability of neuronal cells to alphavirus-induced apoptosis and emphasize the relevance of apoptosis-regulatory mechanisms in shaping host-pathogen interactions in the CNS.

The gene expression data presented here are particularly noteworthy, as virus-induced innate immune responses have not been extensively characterized in the context of SINV infection—especially in neuronal cell models. Although such mechanisms have been more thoroughly described for other alphaviruses, it is reasonable to hypothesize the activation of similar antiviral signaling pathways. Viral RNA species act as pathogen-associated molecular patterns (PAMPs) and are detected by host PRRs, including endosomal Toll-like receptors (e.g., TLR3 and TLR7) and cytosolic RLRs such as RIGI and MDA5. These PRRs reside in distinct cellular compartments and exhibit ligand-specific recognition profiles. TLR3 predominantly senses dsRNA, a replication intermediate of many RNA viruses, while TLR7 detects ssRNA, a typical feature of alphavirus genomes. In parallel, cytoplasmic RIGI and MDA5 recognize viral RNA structures and mediate the induction of type-I IFN-s and pro-inflammatory CKs. Upon recognition of viral PAMPs, PRRs activate downstream signaling cascades involving adaptor proteins, ultimately leading to transcriptional activation of antiviral and immunoregulatory genes [1,4,5]. This complex innate immune network plays a critical role in shaping the host response and determining neuronal cell fate during SINV infection. In our study, the early upregulation of *PRR*s—observed as soon as 3 to 6 hpi—suggests their critical and indispensable involvement in the initial detection of SINV-derived viral components in SH-SY5Y neuroblastoma cells (Figure 6). Specifically, the mRNA expression levels of endosomal *TLR3* and *TLR7* were already elevated at 3 hpi (Figure 6C,D), whereas the cytosolic RNA sensors *RIGI* and *MDA5* showed a distinguished increase by 6 h (Figure 6A,B), relative to uninfected controls. These findings strongly support the idea that both endosomal and cytosolic PRRs play complementary and indisputable essential roles in sensing SINV in SH-SY5Y cells. Consistent with innate immune responses observed in other alphaviruses, this early *PRR* activation is likely to trigger downstream antiviral pathways. This includes the induction of type-I IFNs and pro-inflammatory mediators, thereby initiating a robust cellular response aimed at restricting viral replication. IFN-β is a pivotal component of the type-I IFN response and exerts potent antiviral effects during the early stages of infection. Following recognition of viral RNA by PRRs, downstream signaling cascades lead to the activation of transcription factors such as *IRF3* and *IRF7*, which are crucial for the induction of *IFN-β* expression. Once secreted, *IFN-β* orchestrates a broad antiviral response by regulating the transcription of numerous CKs and chemokines that facilitate the recruitment and activation of innate and adaptive immune cells, including NK cells and T lymphocytes. This machinery contributes to the localization and elimination of the infection [1,32]. Previous studies have demonstrated that C7/10 SINV infection in HEK-293 and BHK-21 cells induces robust augmentation of *IFN-β* and *interferon-stimulated genes* (*ISGs*) as part of the antiviral response [32]. In our neuronal model, we observed a marked increase in *IFN-β* mRNA expression at 16 hpi, followed by a gradual decline. Nevertheless, *IFN-β* transcript levels remained consistently higher than those detected in uninfected control cells throughout the infection course (Figure 7A). These findings suggest that SH-SY5Y cells, despite their neuronal origin, can mount a functional type-I IFN response. This response may help restrict viral replication and reduce the severity of virus-induced cytopathology. In the context of the CNS, IFN-β may play a protective role by attenuating viral spread and reducing neuronal cell death. The sustained expression of *IFN-β* observed in our study (Figure 7A) supports the hypothesis that neurons can contribute to local antiviral immunity and highlights their potential role in neuroimmune interactions during SINV infection. The observed increase in mRNA levels of pro-inflammatory CKs—*IL-1β*, *IL-6*, and *TNF-α*—between 12 and 16 hpi likely reflects an early host defense response to SINV (Figure 7B–E). However, by 30 h, the expression of these CKs had substantially declined and most of them remained elevated compared to the corresponding controls. (Figure 7B–E). This trend may indicate the termination of the inflammatory response at the transcriptional level. Nevertheless, further studies are warranted to evaluate CKs at the protein level, as post-transcriptional and translational mechanisms may also influence the immune landscape. A similar immune activation profile has been reported during CHIKV infection, where viral challenge triggered a strong innate immune response characterized by elevated production of pro-inflammatory mediators [33]. While these inflammatory signals are essential for antiviral defense, their dysregulation—particularly in the context of the CNS—can be detrimental. Uncontrolled neuroinflammation may contribute to neuronal dysfunction or death, emphasizing the critical necessity for tight regulation of immune responses in neural tissues [34]. IL-10 is a crucial anti-inflammatory CK that plays a multifaceted role in modulating immune responses. One of its primary functions is the suppression of macrophage activation and the downregulation of pro-inflammatory CK production, thereby exerting immunosuppressive effects that can mitigate tissue damage caused by excessive inflammation [35]. Similar findings have been reported in cases of CHIKV-induced arthritis, in which IL-10 appears to play a protective role by reducing inflammatory damage [34,35]. It is plausible that IL-10 also functions in a regulatory capacity during SINV infection. Our study found that the early induction of *IL-10* mRNA in SH-SY5Y neuroblastoma cells occurred following exposure to SINV, with transcript levels gradually declining over the course of the infection (Figure 7D). The role of IL-10 in neurotropic alphavirus infections is particularly intricate, as it must balance the dual objectives of protecting neural tissue while still allowing for an effective antiviral response. In the CNS, IL-10 is able to reduce neuroinflammation primarily by inhibiting the excessive activation of microglia and astrocytes, as well as by suppressing the production of key pro-inflammatory CKs, including TNF-α and IL-1β. This immunoregulatory function helps mitigate neuronal injury and preserve tissue integrity. However, IL-10 also suppresses T cell and macrophage activity within the CNS, which may inadvertently permit sustained viral replication and persistence. This could potentially contribute to the development of latent or chronic infections [33,34]. β-Catenin is a central effector of the canonical Wnt/β-catenin signaling pathway that functions as a multifaceted cellular regulator. Beyond its well-established roles in cell fate determination and developmental processes, β-catenin is increasingly recognized as a modulator of immune responses and viral replication [36]. During a viral infection, β-catenin can influence the expression of inflammatory and antiviral genes. Its effects on type-I IFN signaling are context-dependent, either enhancing or suppressing the response, depending on the pathogen and host cell type. In specific viral infections, such as HIV and HBV, β-catenin activation has been associated with anti-inflammatory effects and reduced virus-induced cytopathogenicity, suggesting a potential protective or regulatory role in limiting host tissue damage. Conversely, in certain contexts, β-catenin activation may support viral persistence and replication by attenuating host immune responses. Moreover, β-catenin signaling exhibits anti-apoptotic properties, potentially supporting the survival of infected cells and thereby enhancing viral propagation. Interestingly, a previous study demonstrated downregulation of β-catenin at the protein level following CHIKV infection. This finding only partially aligns with prior observations regarding other viral pathogens [37]. This highlights the virus-specific modulation of β-catenin and underscores the need for further investigation. Accordingly, future studies could focus on assessing β-catenin expression at the protein level, as current literature suggests that its regulation may differ substantially even among closely related alphaviruses [38,39]. Interestingly, changes in the expression levels of regulatory molecules such as *IL-10* (Figure 7D) and *β-catenin* (Figure 7F) mRNA suggest that SINV not only initiates a specific antiviral immune response but also modulates intrinsic cellular regulatory pathways. Although both molecules may potentially support cell survival, the cellular balance appears to shift toward apoptotic cell death by 36 hpi, even at low viral loads (based on our observations). This dynamic temporal shift reflects a complex interplay between antiviral defense mechanisms and virus-induced cellular stress responses. These findings may enhance our understanding of the intricate pathogenesis of SINV, although further studies are still required, particularly, at the protein level.

Nonetheless, an important limitation of this study is that it only analyzed one SINV strain. It has been hypothesized that different SINV strains may have varying levels of neuroinvasive capacity and pathogenicity, including their ability to breach the blood–brain barrier and cause neurological disease. Therefore, while our data provide insights into the mechanisms of SINV–host interactions in neuronal-like cells, they may not fully reflect the biological diversity observed across different SINV strains. Future comparative studies employing strains with distinct neurovirulence profiles are essential for validating and extending these findings. Furthermore, although in vivo mouse models of SINV infection have provided substantial knowledge on viral neuropathogenesis [9,21,40] the present study was deliberately designed as an in vitro investigation using human neuronal cells. Compared to animal models, research employing human-derived neuronal systems remains scarce. Therefore, our findings provide valuable additional insights into SINV–host interactions from a human cellular perspective. However, future studies incorporating animal models are essential for validating these results in vivo and linking molecular mechanisms with neurological outcomes.

In summary, our findings demonstrate that this SINV strain not only induces morphological and apoptotic alterations in SH-SY5Y cells but also triggers complex immunologically relevant pathways that contribute to both antiviral defense and the progression of programmed cell death. Understanding the interplay between apoptotic mechanisms and the accompanying molecular responses may offer deeper insight into SINV-induced neurodegenerative damage. Comprehensive in vitro analyses of SINV and elucidation of its infection dynamics are essential prerequisites for in vivo studies, the identification of potential therapeutic targets, and the development of effective intervention strategies. Moreover, the application of the UV-inactivated virus represents a valuable tool to facilitate such investigations under safer laboratory conditions.

## 5. Conclusions

This study provides novel insights into the cellular responses of human neuroblastoma SH-SY5Y cells to SINV infection, emphasizing both morphological alterations and molecular signaling events that influence neuronal cell fate. We demonstrate that SINV efficiently replicates in these cells, leading to apoptosis through a caspase-dependent pathway characterized by increased expression of caspase-3 and Bax, along with reduced Bcl-2 levels. In parallel, the virus triggers a rapid innate immune response, marked by the early upregulation of PRRs (*TLR3*, *TLR7*, *RIGI*, and *MDA5*), followed by the induction of *IFN-β* and pro-inflammatory CKs such as *IL-1β*, *IL-6*, and *TNF-α*. Notably, *IL-10* and *β-catenin*—both of which are associated with regulatory and anti-inflammatory functions—also exhibited altered expression, suggesting that SINV may modulate immune and survival pathways to influence infection outcome. However, these compensatory responses appear to be hampered by the progressive activation of pro-apoptotic signaling, ultimately leading to neuronal death in vitro even under low viral burden. Taken together, these results illustrate the complex interplay between antiviral defense mechanisms and virus-driven apoptosis in neuronal-like cells. Understanding this balance is critical, as dysregulated immune responses in the CNS may exacerbate viral pathogenesis. Our findings also underscore the importance of in vitro human models in dissecting neurotropic viral infections and demonstrate the utility of UV-inactivated SINV as a safe research tool for downstream studies in lower biosafety environments.

## Data Availability

The data presented in this study are available in the article. Further inquiries regarding the datasets can be directed to the corresponding author.

## Supplementary Materials

The following supporting information can be downloaded at: https://www.mdpi.com/article/10.3390/v17101346/s1, Figure S1: Assessment of cytopathic effects in SH-SY5Y cells following SINV infection by light microscopy; Figure S2: Detection of double-stranded RNA (dsRNA) in SINV-infected SH-SY5Y cells using confocal microscopy; Figure S3: Detection of caspase-3-positive cells by fluorescence microscopy; Figure S4: Detection of Bcl-2-positive cells by fluorescence microscopy; Figure S5: Immunofluorescent detection of Bax-positive cells by fluorescence microscopy. Table S1: Primer sequences applied in this study.

## Abbreviations

The following abbreviations are used in this manuscript:

Ab	Antibody
BSA	Bovine serum albumin
BSL-2	Biosafety level-2
CHIKV	Chikungunya virus
CK	Cytokine
CNS	Central nervous system
CPE	Cytopathic effect
DC	Dendritic cell
dsRNA	Double-stranded RNA
FFU	Focus-Forming Unit
hpi	Hour of post infection
IFN	Interferon
IRF	Interferon regulatory factor
ISG	Interferon stimulated gene
MDA5	Melanoma differentiation-associated protein 5
MEM	Minimum Essential Media
NK	Natural killer
PAMP	Pathogen-associated molecular pattern
PBS	Phosphate buffered saline
PRR	Pattern recognition receptor
RIG	Retinoic acid-inducible gene
RLR	RIG-I-like receptor
SINV	Sindbis virus
ssRNA	Single-stranded RNA
TCID50	Tissue culture infectious dose 50
TLR	Toll-like receptor
UV	Ultraviolet radiation
Wnt	Wingless related integration site

## References

[1] Carpentier K.S., Morrison T.E. (2018). Innate immune control of alphavirus infection. Curr. Opin. Virol..

[2] Rulli N.E., Melton J., Wilmes A., Ewart G., Mahalingam S. (2007). The molecular and cellular aspects of arthritis due to alphavirus infections: Lesson learned from Ross River virus. Ann. N. Y. Acad. Sci..

[3] Adouchief S., Smura T., Sane J., Vapalahti O., Kurkela S. (2016). Sindbis virus as a human pathogen-epidemiology, clinical picture and pathogenesis. Rev. Med. Virol..

[4] Takeuchi O., Akira S. (2007). Recognition of viruses by innate immunity. Immunol. Rev..

[5] Guerrero-Arguero I., Tellez-Freitas C.M., Weber K.S., Berges B.K., Robison R.A., Pickett B.E. (2021). Alphaviruses: Host pathogenesis, immune response, and vaccine & treatment updates. J. Gen. Virol..

[6] Lum F.M., Ng L.F. (2015). Cellular and molecular mechanisms of chikungunya pathogenesis. Antiviral. Res..

[7] Lombardi Pereira A.P., Suzukawa H.T., do Nascimento A.M., Bufalo Kawassaki A.C., Basso C.R., Dos Santos D.P., Damasco K.F., Machado L.F., Amarante M.K., Ehara Watanabe M.A. (2019). An overview of the immune response and Arginase I on CHIKV immunopathogenesis. Microb. Pathog..

[8] Anderson D., Neri J.I.C.F., Souza C.R.M., Valverde J.G., De Araújo J.M.G., Nascimento M.D.S.B., Branco R.C.C., Arrais N.M.R., Lassmann T., Blackwell J.M. (2021). Zika Virus Changes Methylation of Genes Involved in Immune Response and Neural Development in Brazilian Babies Born with Congenital Microcephaly. J. Infect. Dis..

[9] Lewis J., Wesselingh S.L., Griffin D.E., Hardwick J.M. (1996). Alphavirus-induced apoptosis in mouse brains correlates with neurovirulence. J. Virol..

[10] Cheng E.H., Kirsch D.G., Clem R.J., Ravi R., Kastan M.B., Bedi A., Ueno K., Hardwick J.M. (1997). Conversion of Bcl-2 to a Bax-like death effector by caspases. Science.

[11] Lundstrom K. (2017). Alphavirus-Based Vaccines. Methods Mol. Biol..

[12] Valero Y., Mokrani D., Chaves-Pozo E., Arizcun M., Oumouna M., Meseguer J., Esteban M.Á., Cuesta A. (2018). Vaccination with UV-inactivated nodavirus partly protects European sea bass against infection, while inducing few changes in immunity. Dev. Comp. Immunol..

[13] Sane J., Kurkela S., Levanov L., Nikkari S., Vaheri A., Vapalahti O. (2012). Development and evaluation of a real-time RT-PCR assay for Sindbis virus detection. J. Virol. Methods.

[14] Kopasz Z., Leiner K., Papp H., Lőrincz E., Sipos-Szabó L., Bodó K., Szabó E., Madai M., Zana B., Bajusz D. (2025). Novel semisynthetic glycopeptide antibiotics with antiviral activity against Zika virus and other emerging viruses. Eur. J. Med. Chem..

[15] Bodó K., Boros Á., Rumpler É., Molnár L., Böröcz K., Németh P., Engelmann P. (2019). Identification of novel lumbricin homologues in *Eisenia andrei* earthworms. Dev. Comp. Immunol..

[16] Bodó K., Kellermayer Z., László Z., Boros Á., Kokhanyuk B., Németh P., Engelmann P. (2021). Injury-Induced Innate Immune Response During Segment Regeneration of the Earthworm, *Eisenia andrei*. Int. J. Mol. Sci..

[17] Jansen S., Lühken R., Helms M., Pluskota B., Pfitzner W.P., Oerther S., Becker N., Schmidt-Chanasit J., Heitmann A. (2022). Vector Competence of Mosquitoes from Germany for Sindbis Virus. Viruses.

[18] Hesson J.C., Lundin E., Lundkvist Å., Lundström J.O. (2019). Surveillance of mosquito vectors in Southern Sweden for Flaviviruses and Sindbis virus. Infect. Ecol. Epidemiol..

[19] Ziegler U., Fischer D., Eiden M., Reuschel M., Rinder M., Müller K., Schwehn R., Schmidt V., Groschup M.H., Keller M. (2019). Sindbis virus- a wild bird associated zoonotic arbovirus circulates in Germany. Vet. Microbiol..

[20] Lundström J.O., Hesson J.C., Schäfer M.L., Östman Ö., Semmler T., Bekaert M., Weidmann M., Lundkvist Å., Pfeffer M. (2019). Sindbis virus polyarthritis outbreak signalled by virus prevalence in the mosquito vectors. PLoS Negl. Trop. Dis..

[21] Kimura T., Griffin D.E. (2003). Extensive immune-mediated hippocampal damage in mice surviving infection with neuroadapted Sindbis virus. Virology.

[22] Bergqvist J., Forsman O., Larsson P., Näslund J., Lilja T., Engdahl C., Lindström A., Gylfe Å., Ahlm C., Evander M. (2015). Detection and isolation of Sindbis virus from mosquitoes captured during an outbreak in Sweden, 2013. Vector Borne Zoonotic Dis..

[23] Uejio C.K., Kemp A., Comrie A.C. (2012). Climatic controls on West Nile virus and Sindbis virus transmission and outbreaks in South Africa. Vector. Borne Zoonotic Dis..

[24] Suvanto M.T., Uusitalo R., Otte Im Kampe E., Vuorinen T., Kurkela S., Vapalahti O., Dub T., Huhtamo E., Korhonen E.M. (2022). Sindbis virus outbreak and evidence for geographical expansion in Finland, 2021. Eurosurveillance.

[25] Gutiérrez-López R., Ruiz-López M.J., Ledesma J., Magallanes S., Nieto C., Ruiz S., Sanchez-Peña C., Ameyugo U., Camacho J., Varona S. (2024). First isolation of the Sindbis virus in mosquitoes from southwestern Spain reveals a new recent introduction from Africa. One Health.

[26] Mathew A.M., Mun A.B., Balakrishnan A. (2018). Ultraviolet Inactivation of Chikungunya Virus. Intervirology.

[27] Gracheva A.V., Korchevaya E.R., Ammour Y.I., Smirnova D.I., Sokolova O.S., Glukhov G.S., Moiseenko A.V., Zubarev I.V., Samoilikov R.V., Leneva I.A. (2022). Immunogenic properties of SARS-CoV-2 inactivated by ultraviolet light. Arch. Virol..

[28] Griffin D.E., Hardwick J.M. (1997). Regulators of apoptosis on the road to persistent alphavirus infection. Annu. Rev. Microbiol..

[29] Nava V.E., Rosen A., Veliuona M.A., Clem R.J., Levine B., Hardwick J.M. (1998). Sindbis virus induces apoptosis through a caspase-dependent, CrmA-sensitive pathway. J. Virol..

[30] Salah-eldin A., Inoue S., Tsuda M., Matsuura A. (2000). Abnormal intracellular localization of Bax with a normal membrane anchor domain in human lung cancer cell lines. Jpn. J. Cancer Res..

[31] Hoetelmans R., van Slooten H.J., Keijzer R., Erkeland S., van de Velde C.J., Dierendonck J.H. (2000). Bcl-2 and Bax proteins are present in interphase nuclei of mammalian cells. Cell Death Differ..

[32] Crawford J.M., Buechlein A.M., Moline D.A., Rusch D.B., Hardy R.W. (2023). Host Derivation of Sindbis Virus Influences Mammalian Type I Interferon Response to Infection. Viruses.

[33] Caglioti C., Lalle E., Castilletti C., Carletti F., Capobianchi M.R., Bordi L. (2013). Chikungunya virus infection: An overview. New Microbiol..

[34] Kedzierski L., Tan A.E.Q., Foo I.J.H., Nicholson S.E., Fazakerley J.K. (2022). Suppressor of Cytokine Signalling 5 (SOCS5) Modulates Inflammatory Responses during Alphavirus Infection. Viruses.

[35] Rowell J.F., Griffin D.E. (1999). The inflammatory response to nonfatal Sindbis virus infection of the nervous system is more severe in SJL than in BALB/c mice and is associated with low levels of IL-4 mRNA and high levels of IL-10-producing CD4+ T cells. J. Immunol..

[36] Bodó K., Boros Á., da Costa C.B., Tolnai G., Rumpler É., László Z., Nagyeri G., Németh P., Kille P., Molnár L. (2025). A novel beta-catenin homologue from the earthworm *Eisenia andrei*: Identification and characterization during embryonic development, segment regeneration, and immune response. Int. J. Biol. Macromol..

[37] Chatterjee S., Ghosh S., Datey A., Mahish C., Chattopadhyay S., Chattopadhyay S. (2023). Chikungunya virus perturbs the Wnt/β-catenin signaling pathway for efficient viral infection. J. Virol..

[38] Hillesheim A., Nordhoff C., Boergeling Y., Ludwig S., Wixler V. (2014). β-catenin promotes the type I IFN synthesis and the IFN-dependent signaling response but is suppressed by influenza A virus-induced RIG-I/NF-κB signaling. Cell Commun. Signal..

[39] Jimenez O.A., Narasipura S.D., Barbian H.J., Albalawi Y.A., Seaton M.S., Robinson K.F., Al-Harthi L. (2021). β-Catenin Restricts Zika Virus Internalization by Downregulating Axl. J. Virol..

[40] Jackson A.C., Moench T.R., Trapp B.D., Griffin D.E. (1988). Basis of neurovirulence in Sindbis virus encephalomyelitis of mice. Lab. Investig..

