# Supplementary material for: Sindbis Virus–Host Interactions in Human Neuroblastoma Cells: Implications for Viral Pathogenesis and Replication
Source: Viruses. 2025 Oct 7;17(10):1346. doi: 10.3390/v17101346 (PMC12567755; doi:10.3390/v17101346)
Supplement: Supplementary file 1 [file viruses-17-01346-s001.zip › viruses-3896839-supplementary.pdf]

Supplementary materials

Sindbis Virus–Host Interactions in Human Neuroblastoma Cells: Implications for Viral Pathogenesis and Replication

Kornélia Bodó [1], Zoltán Kopasz [1], Viktória Nyári [1], Krisztina Leiner[1], Péter Engelmann[2], Brigitta Zana[1], Roland Hetényi[1,3], Dániel Hanna[1,3], Krisztián Bányai[4,5], Mónika Madai[1], Gréta Varga[1], and Anett Kuczmog [1,6]*

[1]National Laboratory of Virology, Szentágothai Research Center, University of Pécs, H-7624 Pécs, Hungary; bodo.kornelia@pte.hu (K.Bodó); kopasz.zoltan@pte.hu (K.Z.); nyari.viktoria@pte.hu (V.NY.); leiner.krisztina@pte.hu (K.L.); zana.brigitta@pte.hu (B.Z.); roland.hetenyi@aok.pte.hu (R.H.); daniel.hanna@aok.pte.hu (D.H.); bkrota@hotmail.com (K.Bányai); madai.monika@pte.hu (M.M.); varga.greta@pte.hu (V.G.); kuczmog.anett@pte.hu (K.A.)

[2]Department of Immunology and Biotechnology, Medical School, Clinical Center, University of Pécs, H-7624 Pécs, Hungary; engelmann.peter@pte.hu (P.E.)

[3]RoLink Biotechnology Kft., Szentágothai Research Center, University of Pécs, H-7624 Pécs, Hungary; roland.hetenyi@aok.pte.hu (R.H.); daniel.hanna@aok.pte.hu (D.H.)

[4]Department of Medical Biology, Medical School, University of Pécs, H-7624 Pécs, Hungary; bkrota@hotmail.com

[5] Department of Pharmacology and Toxicology, University of Veterinary Medicine, H-1078 Budapest, Hungary

[6]Institute of Biology, Faculty of Sciences, Department of Molecular Biology and Microbiology, University of Pécs, H-7624 Pécs, Hungary; kuczmog.anett@pte.hu (K.A.)

*****Correspondence: kuczmog.anett@pte.hu (K.A.)

1. FIGURES

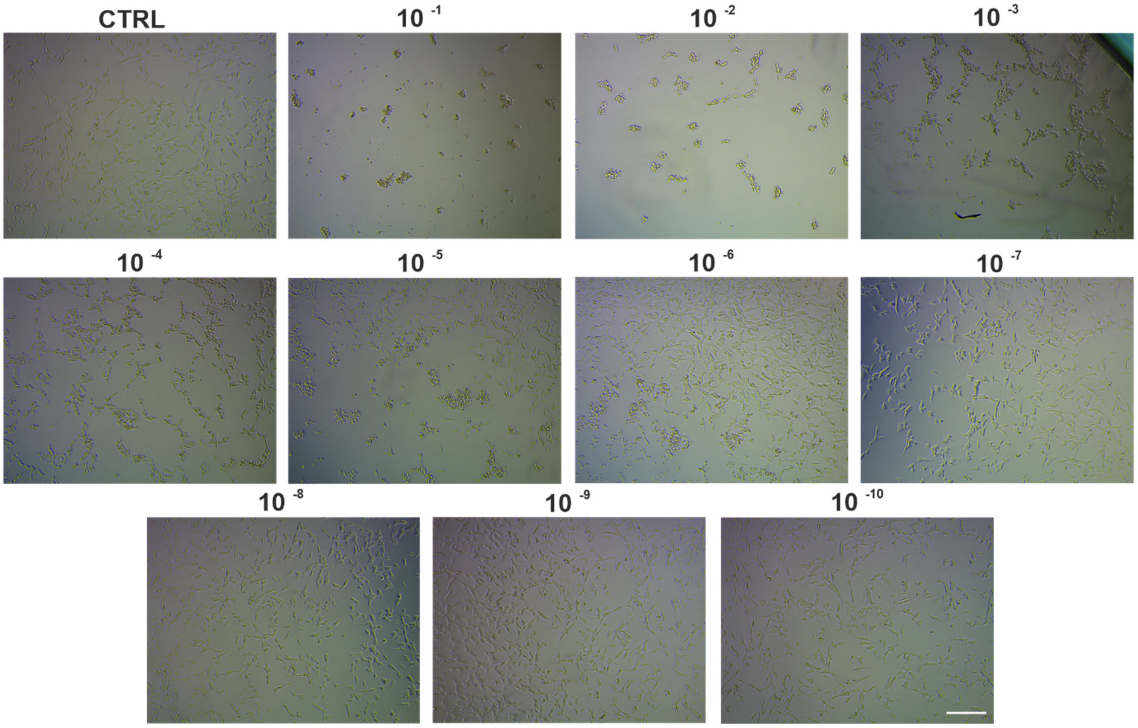

Figure S1. Assessment of cytopathic effects in SH-SY5Y cells following SINV infection by light microscopy. Representative light microscopic images of SH-SY5Y neuroblastoma cells 24 h after infection with SINV, corresponding to samples used for $TCID_{50}$ titer determination. Morphological alterations consistent with viral cytopathicity were observed. Scale bar: 100 µm.

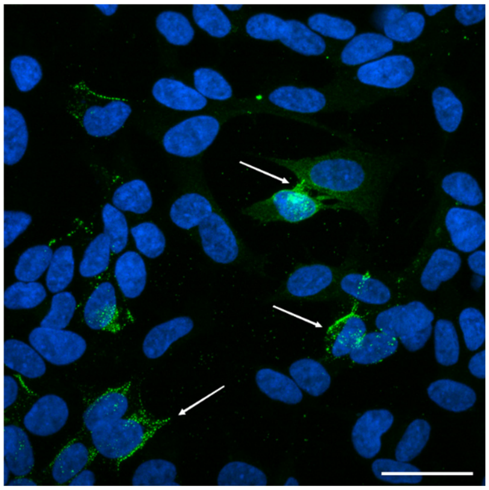

Figure S2. Detection of double-stranded RNA (dsRNA) in SINV-infected SH-SY5Y cells using confocal microscopy. Immunofluorescent staining of viral dsRNA in SH-SY5Y neuroblastoma cells infected with SINV at an MOI of 0.001, 24 hours post-infection. The staining highlights active viral replication intermediates. Samples were also used in parallel UV-C inactivation experiments to assess replication suppression. Images were acquired using confocal microscopy. Scale bar: 20 µm.

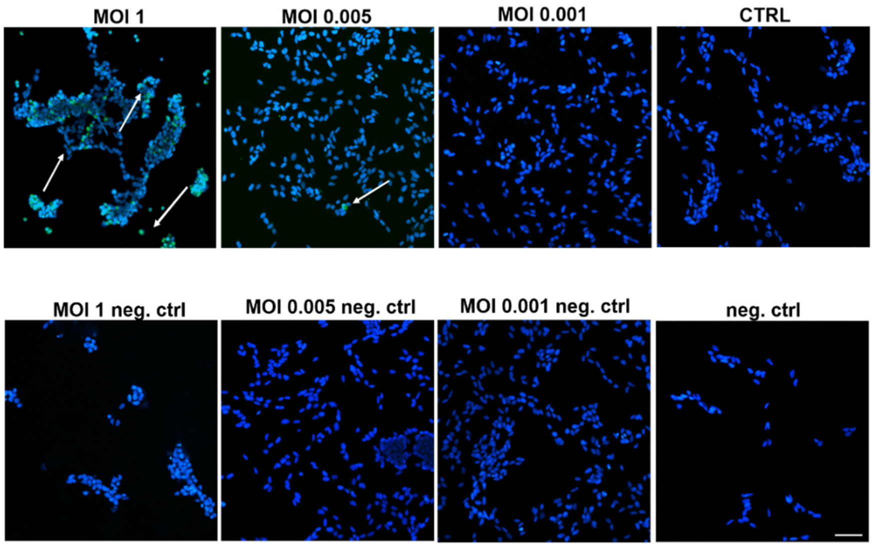

Figure S3. Detection of caspase-3-positive cells by fluorescence microscopy. Immunofluorescent localization of the apoptotic marker caspase-3 in SH-SY5Y neuroblastoma cells 24 hpi. The cells were either uninfected (CTRL) or infected with SINV at MOIs of 0.001, 0.005, or 1. Arrows in the upper panel indicate caspase-3-positive apoptotic cells. The lower panel shows the same exposures without AB-labeling. Scale bar: 50 µm

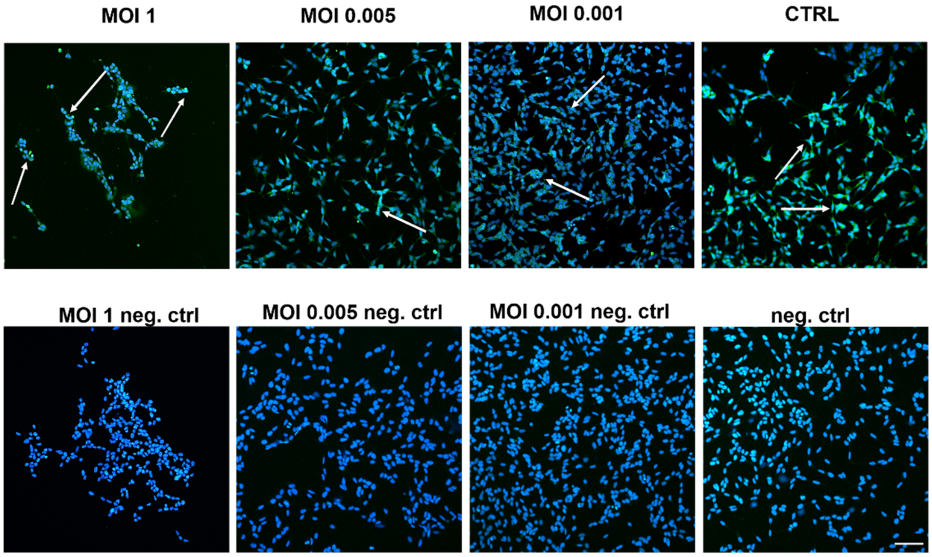

Figure S4. Detection of Bcl-2-positive cells by fluorescence microscopy. Immunofluorescent visualization of the anti-apoptotic marker Bcl-2 in SH-SY5Y neuroblastoma cells 24 hpi. The cells were either uninfected (CTRL) or infected with SINV at MOIs of 0.001, 0.005, or 1. Arrows in the upper panel indicate Bcl-2-positive cells. The lower panel shows the same cells without antibody labeling and serves as an unstained control. Scale bars: 50 µm.

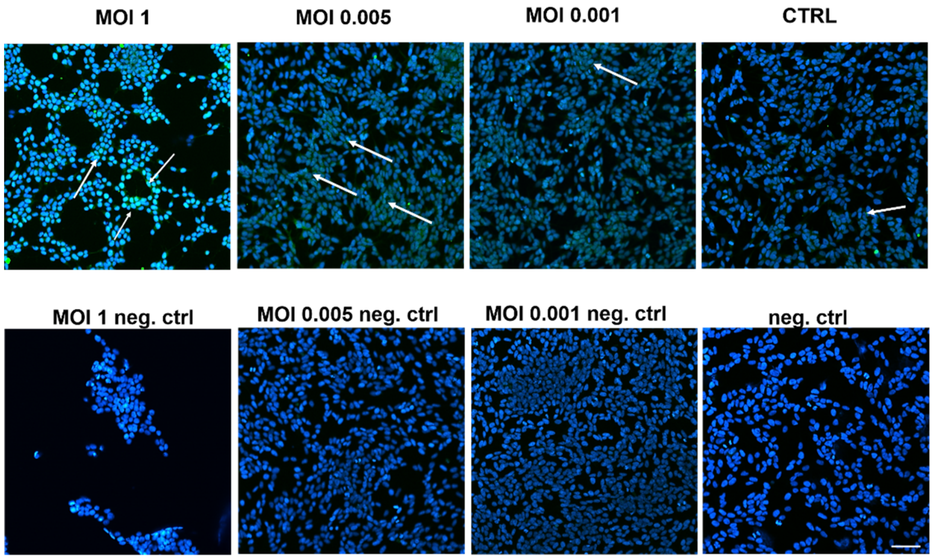

Figure S5. Immunofluorescent detection of Bax-positive cells by fluorescence microscopy. Immunofluorescent localization of the pro-apoptotic marker Bax in SH-SY5Y neuroblastoma cells 24 hpi. The cells were either uninfected (CTRL) or infected with SINV at MOIs of 0.001, 0.005, or 1. Arrows in the upper panel indicate Bax-positive cells. The lower panel shows the same cells without antibody labeling and serves as an unstained control. Scale bars: 50 µm.

Table S1.

Primer ID	Application	Sequence (5'-3')	Oligo-nucleotide primer size
SINV-NSP1-F	RT-qPCR	TGA TAC TGG TGC GAA AAC A	19 nt
SINV-NSP1-R	RT-qPCR	GGT TCC TAC CAC AGC GAC GAT	19 nt
SINV-P-FAM	RT-qPCR	TTG GAC ATA GGC AGC GCA	18 nt
RPLP0-F	qPCR	GGC CCG AGA AGA CCT CCT T	19 nt
RPLP0-R	qPCR	CGC TGG CTC CCA CTT TGT	18 nt
TLR3-F	qPCR	GCC CTT TGG GAT GCT GTG T	19 nt
TLR3-R	qPCR	ATC ATC GGG TAC CTG AGT CAA CTT	24 nt
TLR-7-F	qPCR	GAT CTG TAC ACC TGT GAG TTA G	22 nt
TLR7-R	qPCR	ATA CCA CAC ATC CCA GAA ATA G	22 nt
RIG1-F	qPCR	CAA CAC CCG TAC AAT ATG ATC ATG T	25 nt
RIG1-R	qPCR	ACA CCA ACC GAG GCA GTC A	19 nt
MDA5-F	qPCR	GGC CAG GGT GAA AAT GTA CAT C	22 nt
MDA5-R	qPCR	GGT GGC GAC TGT CCT CTG AA	20 nt
IL-1β-F	qPCR	TGG AAA TTG CCC TCG TTG TT	20 nt
IL-1β-R	qPCR	TGT CAG CGC ATC AAA AGC AT	20 nt
IL-6-F	qPCR	CCT GAC CCA ACC ACA AAT GC	20 nt
IL-6-R	qPCR	CCT TAA AGC TGC GCA GAA TGA	21 nt
TNF-α-F	qPCR	ACA TCC AAC CTT CCC AAA CG	20 nt
TNF-α-R	qPCR	GCC CCC AAT TCT CTT TTT GAG	21 nt
IL-10-F	qPCR	CTG GGT TGC CAA GCC TTG T	19 nt
IL-10-R	qPCR	AGT TCA CAT GCG CCT TGA TG	20 nt
IFN-β-F	qPCR	GAC ACT GGT CGT GTT GTT GAC A	22 nt

IFN-β-R	qPCR	GCA AGT TGT AGC TCA TGG AAA GAG	24 nt
β-catenin-F	qPCR	TAC CGC TGG GAC CTT GCA	18 nt
β-catenin-R	qPCR	AAT CCA CTG GTG AAC CAA GCA	21 nt

Table S1. Primer sequences applied in study.
